# Time-dependent shifts in microbiota and metabolites of Yuba under ambient storage: implication for quality deterioration and spoilage

**DOI:** 10.3389/fnut.2026.1925390

**Published:** 2026-08-18

**Authors:** Yuan Chen, Hongwei Li

**Affiliations:** School of Life Science, Huizhou University, Huizhou, China

**Keywords:** 16S rRNA gene sequencing, microbial community, spoilage, storage, UPLC–MS/MS, Yuba

## Abstract

**Introduction:**

Yuba (tofu skin), a high-protein and high-lipid plant-based food, is prone to spoilage at ambient temperatures, but the underlying mechanisms are not fully understood. This study aimed to elucidate the dynamic changes in the microbial community and metabolites of Yuba during ambient storage to investigate quality deterioration and identify potential biomarkers for quality control.

**Methods:**

16S rRNA gene sequencing and untargeted LC-MS/MS metabolomics were employed to analyze Yuba samples stored at room temperature (25 ± 2 °C) for four durations: 7 days (FZ), 50 days (C), 105 days (B), and 460 days (A). Microbial DNA extraction, library construction, sequencing, and bioinformatics analysis were performed. Metabolite extraction and LC-MS/MS analysis were conducted, followed by data preprocessing, identification, and statistical and pathway analysis. Correlation analysis was performed between differentially abundant metabolites and microbial genera.

**Results:**

Storage duration significantly influenced the Yuba metabolome, as evidenced by clear separation in Principal Component Analysis (PCA) and Partial Least Squares-Discriminant Analysis (PLS-DA) scores. Glycerophospholipid and fatty acyl metabolism were significantly altered, with increases in LysoPEs, LysoPA, PCs, and fatty acyls observed during prolonged storage. At the microbial level, Firmicutes dominated throughout storage, with a shift towards increasing abundance of Firmicutes and decreasing abundance of Proteobacteria as storage time increased. *Lysinibacillus* emerged as the predominant genus after long-term storage (460 days), showing a significant increase in relative abundance from 0.27% to 52.33%. Specific bacterial genera, notably *Lysinibacillus* and *Macrococcus*, demonstrated significant correlations with metabolite levels, suggesting their crucial roles in shaping the Yuba metabolome. Microbial functional predictions were associated with lipid, amino acid, and nucleotide metabolism. Eleven common pathways were identified between significantly altered microbes and metabolites, including glycerophospholipid metabolism and amino acid biosynthesis.

**Discussion:**

This study provides a systematic perspective on Yuba deterioration during ambient storage, identifying microbial and metabolic signatures correlated with quality changes. The dynamic remodeling of the metabolome, particularly the increase in lipids and amino acid-related metabolites, coupled with the dominance of *Lysinibacillus*, highlights their potential roles in spoilage. The identified microbial and metabolic signatures serve as promising candidates for future validation as biomarkers to extend shelf life and maintain product quality. Further research involving pure culture studies and metatranscriptomics is warranted to confirm causal relationships and elucidate specific mechanisms.

## 1 Introduction

The burgeoning consumer demand for sustainable and health-conscious diets is driving substantial growth in the plant-based protein market. Yuba, a traditional East Asian food product also known as “tofu skin,” is formed as a protein- and lipid-rich film on the surface of simmering soymilk (1). Its appealing texture and favorable nutritional composition (2, 3) have positioned Yuba as a promising plant-based meat alternative. However, its high protein content (approximately 52%) and lipid content (approximately 24%) (3) render Yuba highly susceptible to spoilage during storage. This spoilage manifests as undesirable discoloration, off-flavors, and textural degradation, posing a significant challenge to producers and limiting its market potential.

Lipid oxidation represents a major mechanism underlying Yuba spoilage, particularly involving the oxidation of polyunsaturated fatty acids (PUFAs) such as linoleic acid (C18:2, LA) and *α*-linolenic acid (C18:3, ALA), which constitute the predominant fatty acids in soybeans (4). Lipoxygenase-catalyzed oxidation leads to the generation of characteristic volatile compounds, including (E)-2-octenal and (E, E)-2,4-heptadienal derivatives, which contribute significantly to the aroma profile (5). Conversely, non-enzymatic oxidation results in the formation of hydroperoxides and volatile aldehydes (e.g., hexanal), which are major contributors to off-flavor development (4). Furthermore, soy proteins, especially glycinin and *β*-conglycinin, are vulnerable to reactive oxygen species (ROS)-mediated carbonylation and aggregation, leading to decreased solubility and digestibility (6). Collectively, Yuba spoilage is a complex process resulting from the interplay of physicochemical changes (lipid oxidation, protein degradation) and microbial activity, necessitating a comprehensive systems-level investigation.

Although drying and vacuum packaging are commonly employed preservation techniques, they often fail to completely eliminate microbial contamination, resulting in spoilage during storage (7). The Yuba production process, which typically involves heating soymilk in open vats, inevitably introduces a diverse array of microorganisms from sources such as equipment surfaces (e.g., biofilms formed by *Bacillus* spp.), water, and the surrounding air (8). While Pseudomonas and Enterobacteriaceae are frequently implicated in the spoilage of various food products (9), studies have indicated that *Bacillus* spp. and lactic acid bacteria (LAB) typically dominate the Yuba microbiome, a phenomenon attributed to the heat resistance of *Bacillus* spores and the acid tolerance of LAB, respectively (10). Although LAB (e.g., *Lactobacillus*) can contribute beneficially to food preservation through fermentation, their proteolytic activity in non-fermented Yuba can lead to the formation of undesirable off-flavors via the release of bitter peptides or sulfur-containing compounds (11). Moreover, certain *Bacillus* spp. exhibit probiotic properties (12) and contribute to desirable flavor development in fermented foods, including doenjang (13). However, a comprehensive systems-level analysis correlating microbial community dynamics with metabolomic shifts during the spoilage process in Yuba is currently lacking.

Currently, the intricate interplay between oxidative reactions and microbial metabolism, and their combined influence on the formation of flavor compounds (e.g., aldehydes, ketones, acids, sulfur compounds, peptides, and amino acid derivatives), remains poorly understood. While Bi et al. (11) have identified key volatile aldehydes and alcohols associated with Yuba spoilage, the corresponding precursors (e.g., hydroxylated polyunsaturated fatty acids, sulfur-containing amino acids) and non-volatile metabolites (e.g., dipeptides, organic acids) are still insufficiently characterized. For instance, acetoin derived from LAB or proteolysis mediated by Bacillus may generate umami or bitter notes, similar to those noted in soy sauce fermentation (14). However, a concurrent analysis of microbial succession and non-volatile metabolite dynamics in ambient-stored Yuba has not been performed.

To address these knowledge gaps, this study employed an integrated approach, combining 16S rRNA gene sequencing to characterize the microbial community with LC–MS metabolomics to analyze non-volatile metabolite changes during storage. Metabolomics enables qualitative and quantitative analysis of metabolites at the molecular level, facilitating the detection of dynamic changes in metabolite profiles (15). For instance, GC–MS analysis has identified 1-hexanal-M, 1-hexanal-D, 2-methylbutanal-M, 2-methylbutanal-D, and 1-octen-3-one as key aroma markers in room-temperature-stored Yuba (11). However, LC–MS-based metabolomic changes associated with Yuba storage duration remain unreported. Complementary to this, 16S rDNA amplicon sequencing—a widely applied technique in food analysis (16–19)—was integrated to characterize bacterial communities. We hypothesize that dynamic shifts in microbial consortia—for instance, changes in the relative abundance of specific Bacillus species (e.g., *B. cereus*, *B. subtilis*) and LAB—will correlate with divergent metabolite profiles and distinct patterns of quality deterioration. Specifically, this study aims to: (1) identify microbial taxa and metabolites that serve as predictive biomarkers for Yuba spoilage during storage; (2) investigate the relationship between shifts in the microbial community and alterations in the metabolite profile of Yuba during storage; and (3) identify novel non-volatile biomarkers to improve shelf-life prediction. By establishing these detailed cause-and-effect relationships, this research will yield fundamental insights crucial for developing targeted preservation strategies.

## 2 Materials and methods

### 2.1 Yuba sample preparation and collection

Yuba was produced via interfacial polymerization as described by Wang et al. (3) with modifications. Briefly, soymilk was heated to 85 °C in a shallow pan, and the protein-lipid film that formed on the surface (Yuba) was carefully lifted and dried at 55 °C for 2 h in a forced-air oven. The dried Yuba sheets were then cut into uniform pieces (5 cm x 5 cm) and vacuum-sealed in polyethylene bags (*n* = 32 bags). The samples were stored at room temperature (25 ± 2 °C, monitored by data loggers) for four durations: 7 days (group FZ), 50 days (group C), 105 days (group B), and 460 days (group A). The storage times were selected to capture key stages of Yuba spoilage under realistic ambient conditions. The 7-day sample (FZ) represents a fresh baseline, crucial for understanding the initial state. The 50-day sample (C) was Chosen to capture early microbial and metabolic shifts where spoilage might become organoleptically detectable. This point is critical for identifying early warning signs. The 105-day sample (B) represents an intermediate stage of advanced spoilage, allowing observation of progression. The 460-day sample (A) was included to understand the endpoint of the spoilage process, identifying the ultimate survivors of the microbial community and the final metabolic products, which is crucial for identifying robust biomarkers for long-term quality prediction. All samples were provided by Guangdong Runze Food Co., Ltd. (Guangdong, China). Each group comprised eight biological replicates (n = 8). All 32 samples (8 replicates x 4 groups) originated from the exact same production batch.

### 2.2 Metabolite extraction and LC–MS/MS analysis

Sample Preparation: Approximately 50 mg (±5 mg) of each sample was homogenized in 500 μL of ice-cold 80% methanol using a bead mill. The homogenate was incubated at −20 °C for 30 min to precipitate proteins, followed by centrifugation at 20,000 g for 10 min at 4 °C. The supernatant was transferred to a new vial and centrifuged again for 5 min. The resulting supernatant was collected for UPLC-HRMS analysis. A quality control (QC) sample was prepared by pooling equal aliquots from each sample supernatant.

Chromatography: An ultra-high-performance liquid chromatography system (Vanquish Flex UPLC, Thermo Fisher Scientific) was used for analysis. Metabolites were separated on an ACQUITY UPLC T3 column (100 mm × 2.1 mm, 1.8 μm; Waters). The mobile phase consisted of 5 mM ammonium acetate and 5 mM acetic acid in water (A) and acetonitrile (B). Gradient elution was performed as follows: 0–0.5 min, 1% B; 0.5–6.5 min, 1–99% B; 6.5–8.0 min, 99% B; 8.0–8.5 min, 99–1% B; 8.5–10.0 min, 1% B. The flow rate was 0.35 mL/min, the injection volume was 4 μL, and the column temperature was maintained at 40 °C.

Mass Spectrometry: A high-resolution tandem mass spectrometer (Orbitrap Exploris 120, Thermo Fisher Scientific) was used for metabolite detection. Samples were analyzed in both positive and negative electrospray ionization (ESI) modes. The ESI temperature was 350 °C. The spray voltage was +3.8 kV (positive mode) and −3.4 kV (negative mode). Ion source parameters were as follows: Sweep gas, 1 Arb; Auxiliary gas (Gas 1), 15 Arb; Sheath gas (Gas 2), 50 Arb. Mass spectra were acquired using full scan (m/z 70–1,050, resolution 60,000) and data-dependent acquisition (DDA) modes. The full scan AGC target was set to Standard with Auto maximum injection time (IT). For DDA, the top 4 ions (intensity threshold > 5,000) were selected from each full scan. DDA spectra were acquired at a resolution of 15,000, with Custom AGC target and Custom maximum IT. Dynamic exclusion was enabled (4 s).

### 2.3 Data analysis of LC–MS/MS

Raw MS data were preprocessed using XCMS software (45) for peak picking, alignment, and grouping to extract meaningful features. Raw LC–MS data files were converted to mzXML format and processed using XCMS, CAMERA (20), and metaX (21) toolboxes within the R statistical environment (v4.0). Each detected ion feature was characterized by its retention time (RT) and accurate mass-to-charge ratio (m/z). Peak intensities were recorded, generating a three-dimensional matrix of feature indices (RT-m/z pairs), sample names, and ion intensity information.

#### 2.3.1 Metabolite identification and validation

Metabolite identification was performed by matching the accurate m/z values and retention times of detected features against established metabolite databases, specifically the Kyoto Encyclopedia of Genes and Genomes (KEGG) (22) and the Human Metabolome Database (HMDB) (23). A strict matching criteria of < 5 ppm mass error was applied for m/z matching. While we did not perform targeted MS/MS fragmentation for every detected feature due to the untargeted nature of the study and the large number of detected features, the confidence in identification relies on the accurate mass measurements and database matching. Features that could be confidently matched to known metabolites in these databases were considered identified. Features that could not be unambiguously identified were treated as unknown.

#### 2.3.2 Statistical and pathway analysis

Metabolomic data underwent several processing steps: (1) filtering of features with >80% missing values across samples or >50% missing values in quality control (QC) samples; (2) imputation of missing values using the k-nearest neighbors (KNN) algorithm (24); and (3) data normalization via probabilistic quotient normalization (PQN) (25) to account for variations in sample loading and instrument response.

Cluster heatmaps were generated using the pheatmap R package. Principal component analysis (PCA) and differential metabolite analysis were performed using the metaX R package. Partial least squares-discriminant analysis (PLS-DA) was conducted using the ropls R package (26), and variable importance in projection (VIP) scores were calculated to identify metabolites contributing significantly to group separation. Spearman’s rank correlation coefficients were calculated using the cor R package to assess relationships between metabolites. Differentially abundant metabolites were identified based on the following criteria: *p* < 0.05 (Student’s t-test), fold change > 1.2 or < 0.83, and VIP ≥ 1. KEGG pathway enrichment analysis was performed using a hypergeometric test (*p* < 0.05) to identify significantly overrepresented metabolic pathways. The Short Time-series Expression Miner (STEM) (27) was used to analyze temporal clustering dynamics and assign metabolites to significant time-dependent expression profiles. Network diagrams were constructed based on metabolite-pathway relationships using Cytoscape software (28).

### 2.4 DNA extraction, library construction, and 16S rDNA sequencing

Total microbial DNA was extracted from Yuba samples using the Cetyltrimethylammonium Bromide (CTAB) method (29), a rapid DNA isolation procedure for small amounts of plant tissue. DNA concentration was quantified using a Qubit fluorometer. The V4 region of the 16S rRNA gene was amplified by PCR using primers 341F (5′-CCTACGGGNGGCWGCAG-3′) and 805R (5′-GACTACHVGGGTATCTAATCC-3′). The PCR cycling conditions were: 98 °C for 30 s; 32 cycles of 98 °C for 10 s, 54 °C for 30 s, and 72 °C for 45 s; and a final extension at 72 °C for 10 min. PCR products were purified using AMPure XP Beads and quantified using a Qubit fluorometer. Product quality was assessed using an Agilent 2100 Bioanalyzer and Illumina library quantitative kits (Kapa Biosciences). Libraries were sequenced on an Illumina NovaSeq 6000 platform (paired-end 250 bp) by LC-Bio Technology Co., Ltd. (Hangzhou, China).

### 2.5 Data processing and 16S rDNA sequencing analysis

Sequencing primers were removed from demultiplexed raw sequences using cutadapt (v1.9). Paired-end reads were merged using FLASH (v1.2.8). Low-quality reads (quality score < 20), short reads (< 100 bp), and reads containing > 5% ambiguous bases were filtered out using fqtrim (v0.94). Chimeric sequences were removed using Vsearch (v2.3.4), and the DADA2 pipeline was used for sequence denoising and generation of amplicon sequence variants (ASVs). Species annotation and sequence alignment were performed using the QIIME2 plugin feature-classifier, with the SILVA and NT-16S databases as references. Alpha and beta diversity were calculated using QIIME2, and the relative abundance of bacteria was used for taxonomic analysis. Differentially abundant genera were identified using the Wilcoxon rank-sum test (*p* < 0.05). Linear discriminant analysis effect size (LEfSe) analysis was performed using nsegata-lefse (LDA ≥ 3.0, *p* < 0.05). All other diagrams were generated using R software (v3.4.4).

### 2.6 Correlation analysis of microbiome and metabolomics

Correlation analysis was performed between differentially abundant metabolites and microflora. Differentially abundant metabolites were defined as those with a fold change ≥ 1.2 or ≤ 0.83, *p* < 0.05, and VIP > 1. Differentially abundant genera were defined as those with *p* < 0.05. Pearson correlation coefficients were calculated using MetaboAnalyst (v4.0). Correlation heatmaps illustrating relationships between differentially abundant genera and metabolites were constructed using the cor function in R (18). The Vegan R package was used to perform Procrustes analysis to assess the congruence between microbiome and metabolic profiles (*p* < 0.05). Venn analysis was used to identify common differential pathways (*p* < 0.05).

## 3 Results

### 3.1 Metabolomic profiling reveals storage time-dependent alterations in Yuba

**Figure 1:**
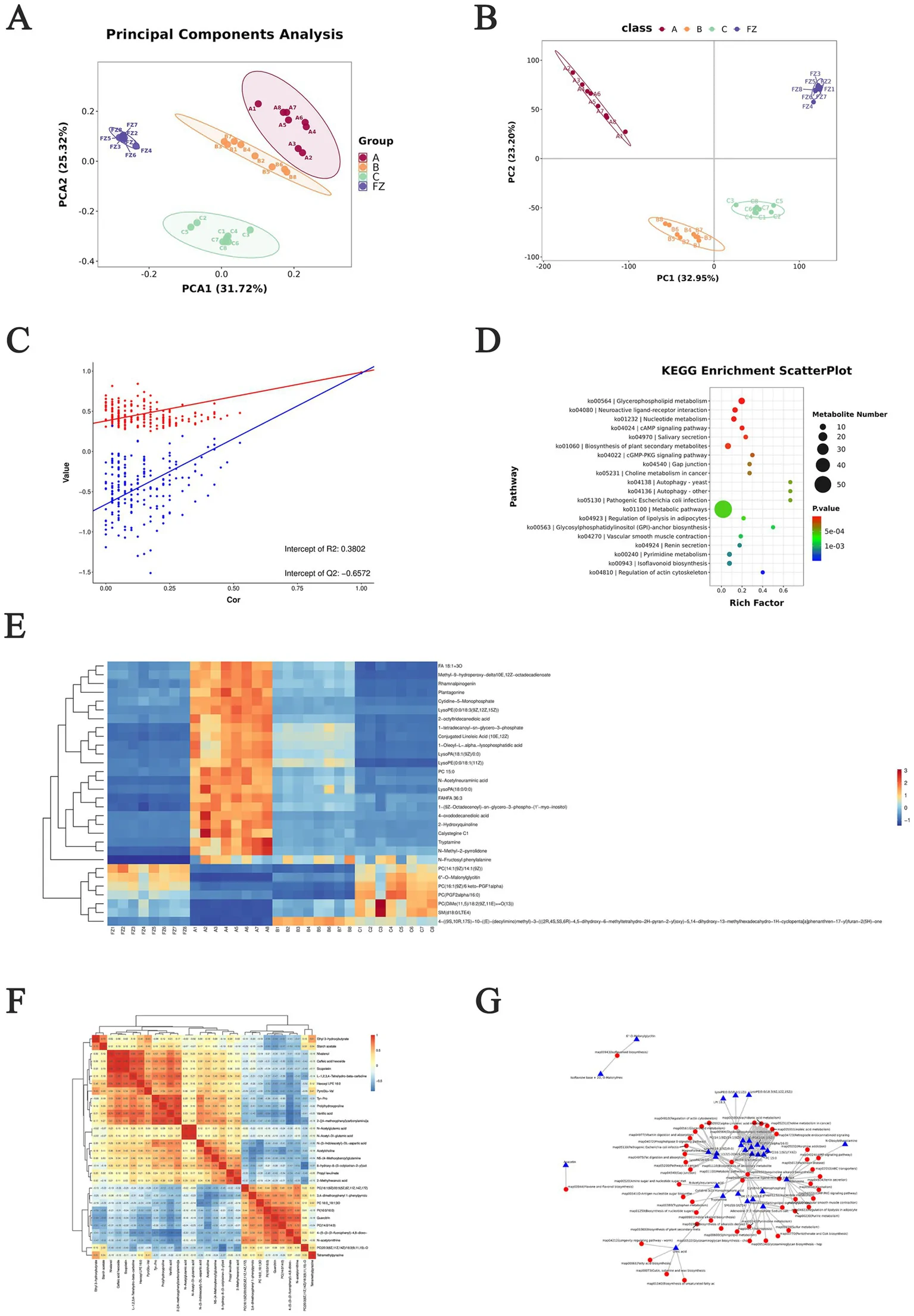
Metabolomic profiling of Yuba during storage reveals dynamic changes in metabolite abundance. **(A)** Principal component analysis (PCA) score plot displaying sample distribution based on metabolomic profiles. Principal component 1 (*PC1*, 31.72% variance) and principal component 2 (*PC2*, 25.32% variance) are shown. Each point represents an individual sample, with colors indicating different storage durations. **(B)** Partial least squares-discriminant analysis (PLS-DA) score plot demonstrating sample separation based on storage duration. Each point represents a sample, with colors indicating different storage durations. **(C)** Permutation testing of the PLS-DA model to assess robustness. *R2* (0.98) and *Q2* (0.97) values are indicated, with regression lines for permuted *Q2* and *R2* values displayed. **(D)** Kyoto Encyclopedia of Genes and Genomes (KEGG) pathway enrichment analysis of differentially abundant metabolites. The *x*-axis represents the -log10(*p*-value) of enrichment, and the *y*-axis represents the Rich Factor. Point size indicates the number of differential metabolites in the pathway, and point color denotes the enrichment *p*-value. **(E)** Hierarchical clustering heatmap of the top 30 differentially abundant metabolites. Red indicates higher abundance, and blue indicates lower abundance. **(F)** Correlation heatmap illustrating Spearman correlation coefficients between the top 30 differentially abundant metabolites. Red indicates positive correlation, and blue indicates negative correlation. **(G)** Network diagram illustrating associations between differentially abundant metabolites and KEGG pathways. Triangles represent metabolites, and circles represent pathways. Lines connect metabolites to their associated pathways.

To investigate the impact of storage duration on the Yuba metabolome, Yuba samples (*n* = 32) were categorized into four groups based on storage duration: FZ (7 days), C (50 days), B (105 days), and A (460 days). Ultra-performance liquid chromatography-mass spectrometry (LC–MS) was employed to analyze the metabolomic profiles of these samples. Unsupervised principal component analysis (PCA) of the metabolomic data (Figure 1A) revealed clear separation among the four storage groups along principal components 1 and 2 (PC1 accounted for 31.72% and PC2 accounted for 25.32%, collectively explaining 57.04% of the variance), demonstrating that storage time significantly alters the Yuba metabolome.

To enhance group separation and identify differential metabolites, a supervised partial least squares-discriminant analysis (PLS-DA) model was employed (Figure 1B). The model demonstrated an explained variance (R^2^Y) of 0.98 and a predictive capability (Q^2^) of 0.97. To evaluate the potential for overfitting, the robustness of the PLS-DA model was confirmed through 200 iterations of 7-fold cross-validation and permutation testing, demonstrating the absence of overfitting (Figure 1C). Consequently, the model was deemed suitable for identifying differential metabolites. The PLS-DA results corroborated the PCA findings, reinforcing the conclusion that distinct metabolic profiles are present in Yuba samples across the four storage stages.

Analysis of variance identified 1,034 metabolites that differed significantly across the four storage groups (FZ, A, B, and C). Using the criteria of variable importance in projection (VIP) > 1 and *p* < 0.05, 324 significantly altered differential metabolites were selected for downstream analysis. Kyoto Encyclopedia of Genes and Genomes (KEGG) pathway enrichment analysis revealed significantly enriched pathways (*p* < 0.05; Figure 1D). The top five pathways were glycerophospholipid metabolism, neuroactive ligand-receptor interaction, nucleotide metabolism, cAMP signaling pathway, and salivary secretion. These pathways span a range of fundamental biological processes, suggesting a coordinated response to physiological stimuli that influences cellular communication, metabolic adaptation, and tissue/organ function.

Next, cluster analysis of the top 30 significantly differentially abundant metabolites identified glycerophospholipids as the most abundant class (12 metabolites), followed by fatty acyls (4 metabolites; Figure 1E). The remaining metabolites were distributed across diverse classes, including sphingolipids, quinolines and their derivatives, anthracenes, organooxygen compounds, pyridines and their derivatives, flavonoids, pyrrolidines, pyrimidine nucleotides, steroids and their derivatives, indoles and their derivatives, carboxylic acids and their derivatives, tropane alkaloids, and unclassified compounds (one metabolite per class). This analysis uncovers a metabolic profile characterized by the dominance of glycerophospholipids (critical for membrane structure and signaling) and fatty acyls (central to energy and lipid metabolism), alongside diverse minor classes that collectively represent a spectrum of functional pathways. These findings not only classify the differentially abundant metabolites but also guide future investigations, thereby prioritizing glycerophospholipid and fatty acyl metabolism for mechanistic studies, while also acknowledging the potential involvement of multiple minor pathways in underlying the biological phenotype.

Specifically, the levels of LysoPE (0:0/18:3(9Z, 12Z, 15Z)), LysoPE (0:0/18:1(11Z)), LysoPA (18:1(9Z)/0:0), PC 15:0, PC (14:1(9Z)/14:1(9Z)), PC (PGF2α/16:0), PC (16:1(9Z)/6keto-PGF1α), and 1-tetradecanoyl-sn-glycero-3-phosphate were significantly elevated during prolonged storage. Similarly, the levels of the four identified fatty acyls exhibited a significant upward trend during storage. Furthermore, the levels of 2-hydroxyquinoline, 4-oxododecanedioic acid, rhamnalpinogenin, conjugated linoleic acid, N-acetylneuraminic acid, tryptamine, N-fructosyl phenylalanine, and calystegine C1 were significantly elevated during storage, whereas the levels of 6”-O-malonylglycitin and SM(d18:0/LTE4) were significantly reduced. These findings demonstrate that prolonged storage induces dynamic changes in the metabolic profile, characterized by widespread increases in lipids, fatty acyls, and diverse non-lipid metabolites, along with selective reductions in specific compounds.

Moreover, correlation analysis uncovered distinct patterns among the 30 significantly differential metabolites (Figure 1F), with many displaying strong positive correlations, indicative of tight functional interactions. Analysis of metabolite-pathway correlations further identified key metabolites involved in multiple pathways (e.g., LysoPA (18:1(9Z)/0:0)) and key pathways containing numerous differential metabolites (e.g., glycerophospholipid metabolism; Figure 1G). This correlation analysis provides crucial insights into the interplay between molecular components and biological processes, confirming the central role of lipid-related components and lipid metabolism processes in the metabolic changes occurring in Yuba during storage.

### 3.2 Temporal trends in metabolite abundance in Yuba during storage

**Figure 2:**
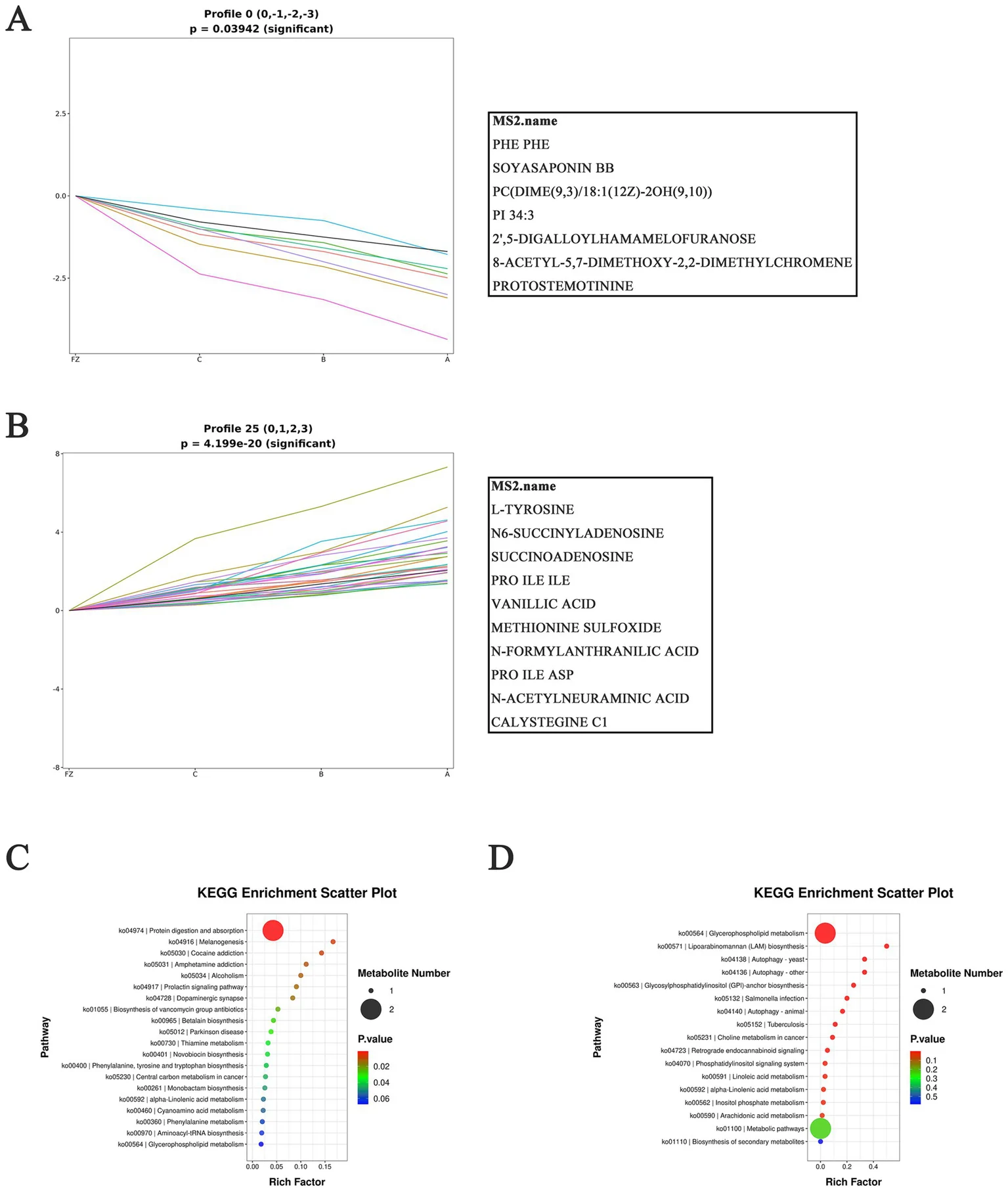
Temporal dynamics of metabolite abundance in Yuba during storage. Short Time-series Expression Miner (STEM) analysis identified significantly enriched temporal profiles of 324 metabolites. Data were normalized to the 7-day group (FZ). Significance was determined by adjusted *p*-value < 0.05. **(A,B)** STEM plots for significantly enriched temporal profiles: Profile #0 (7 metabolites) and Profile #25 (30 metabolites). Each line represents a metabolite exhibiting the indicated trend across storage durations. The black line represents the overall trend for the profile. **(C,D)** KEGG pathway enrichment analysis of metabolites assigned to Profile #0 and Profile #25. The *x*-axis represents the -log10(*p*-value) of enrichment, and the *y*-axis represents the Rich Factor. Point size indicates the number of differential metabolites, and point color denotes the enrichment *p*-value.

To evaluate the temporal dynamics of the metabolome during storage, a short time-series expression miner (STEM) analysis was performed. The 324 significantly differential metabolites were classified based on their abundance changes across the different storage groups. STEM analysis identified two significant temporal profiles, #0 and #25, with respective p -values of 0.03942 and 4.199 × 10^−20^ (Figures 2A,B). Profile #0 showed a steady decrease in abundance with prolonged storage, whereas Profile #25 exhibited a steady increase in abundance as storage duration increased.

Profile #0 consisted of seven metabolites: Phe-Phe, Soyasaponin bb, PC (dime(9,3)/18:1(12z)-2oh (9,10)), PI 34:3, 2′,5-digalloylhamamelofuranose, 8-acetyl-5,7-dimethoxy-2,2-dimethylchromene, and Protostemotinine. Profile #25 primarily contained amino acid (AA)-related metabolites, including L-tyrosine, Pro-Ile-Ile, methionine sulfoxide, Pro-Ile-Asp., N^5^-(4-methoxybenzyl)glutamine, pyroglu-Ile-Arg, *γ*-glutamylleucine, Thr-Ala-Arg, L-allo-isoleucine, and prolylhydroxyproline. Glycerophosphoinositols (e.g., LPI 17:0, LPI 18:3, and 1-(9Z-octadecenoyl)-sn-glycero-3-phospho-(1′-myo-inositol)) were also prominent in this profile. KEGG enrichment analysis of metabolites across profiles revealed significant enrichment of metabolites in Profile #0 for glycerophospholipid metabolism (*p* = 7.313 × 10^−5^) and in Profile #25 for the protein digestion and absorption pathway (*p* = 0.0014; Figures 2C,D). These results suggest that protein and lipid metabolism undergo interconnected and coordinated alterations during Yuba storage, as evidenced by the metabolite profiles and KEGG pathway enrichments.

### 3.3 Shifts in microbial community composition in Yuba during storage

**Figure 3:**
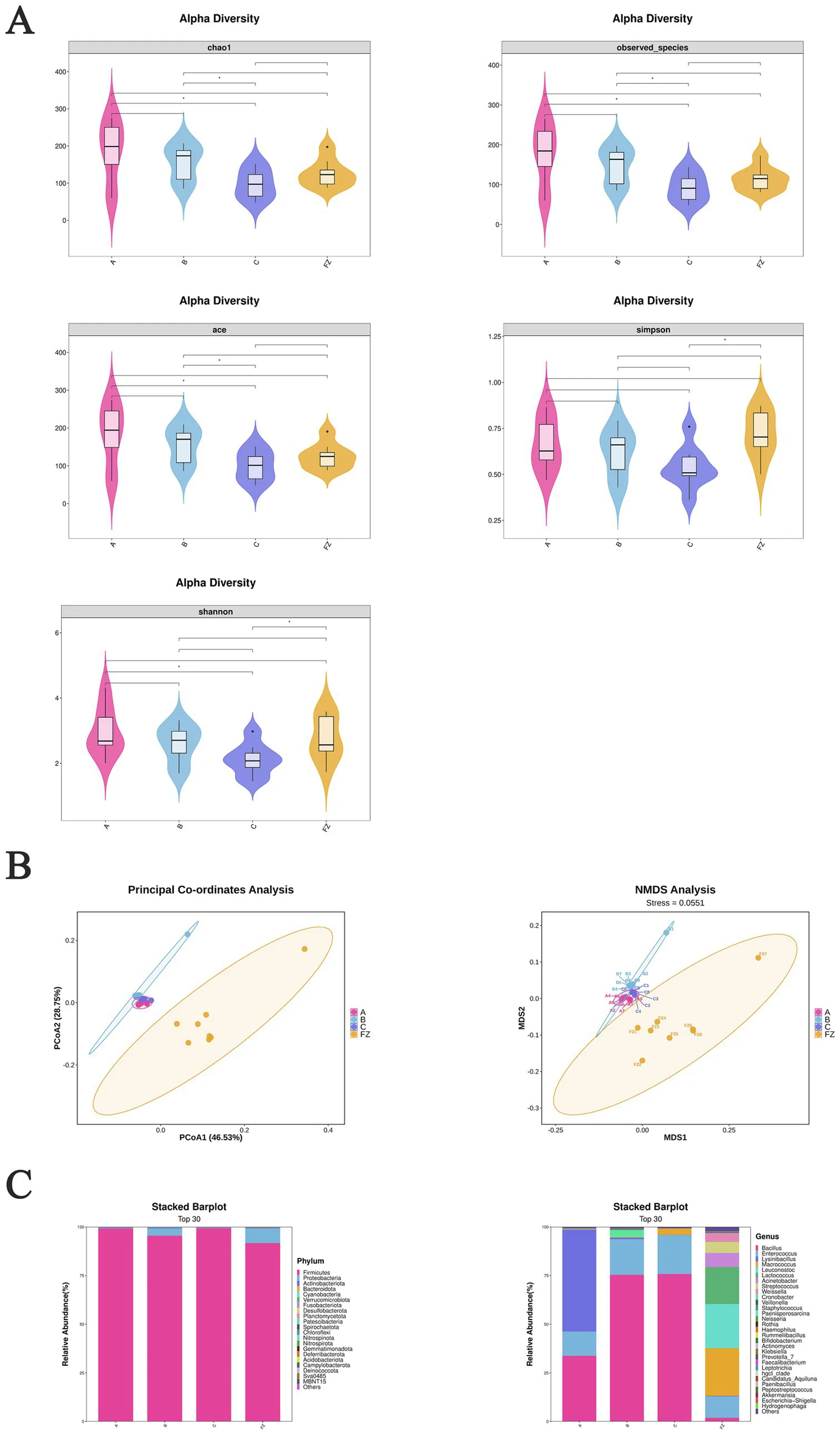
Microbial community structure shifts during Yuba storage. **(A)** Alpha-diversity indices (Ace, Shannon, Simpson, Observed Species, and Chao1) are plotted against storage duration. **(B)** Beta-diversity analysis visualized using principal coordinates analysis (PCoA) and non-metric multidimensional scaling (NMDS). In the PCoA plot, colors represent different storage durations. Variance explained by each principal coordinate is indicated. In the NMDS plot, colors represent different storage durations, and points represent samples. **(C)** Relative abundance of bacterial taxa at the phylum and genus levels across storage durations. The *x*-axis represents storage duration, and the *y*-axis represents relative abundance. Distinct colors represent different phyla or genera.

Variations in the average number of amplicon sequence variants (ASVs) were noted among the groups, with Group A (460 days) harboring the highest ASV count (178), followed by Group B (105 days, 148 ASVs), Group FZ (7 days, 114 ASVs), and Group C (50 days, 92 ASVs), as shown in Figure 3A. Statistical analyses revealed a significant reduction in ASV abundance in Group C compared to Groups A and B (*p* < 0.05), demonstrating the influence of storage duration on ASV abundance. Alpha diversity, as quantified by the Ace and Chao indices (metrics that reflect microbial community richness), further supported the impact of storage duration. Specifically, both the Ace and Chao indices were significantly higher in Groups A and B than in Group C (*p* < 0.05), indicating that microbial community richness is diminished in Group C relative to Groups A and B. These findings demonstrate that storage duration exerts a substantial effect on the microbial community of Yuba. Furthermore, Group C (50 days) emerges as a critical time point, with both ASV abundance and community richness exhibiting significant depletion compared to the longer-stored groups (A and B). In contrast, the shortest-stored group (FZ, 7 days) displayed intermediate ASV levels (114 ASVs) between those of Group C and Group B.

To further investigate variations in microbiota composition, Principal Coordinate Analysis (PCoA) and Non-metric Multidimensional Scaling (NMDS) were employed (Figure 3B). PCoA results, with a cumulative explained variance of 75.28%, revealed no significant differences in microbiota composition among Groups A, B, and C. In contrast, a distinct microbiota composition was observed for Group FZ compared to Groups A, B, and C. NMDS analysis further supported this observation, consistently demonstrating a separation between Group FZ and the other three groups. Taken together, these *β*-diversity analyses demonstrated that prolonged storage (50 to 460 days) did not significantly alter the microbiota composition of Yuba samples (Groups A, B, and C), whereas short-term storage (7 days, Group FZ) resulted in a microbiota profile distinct from the longer-stored groups.

At the phylum level (Figure 3C), Firmicutes was the most dominant phylum across all four groups, with an average relative abundance (RA) of 96.60%, followed by Proteobacteria (average RA: 2.96%) and Actinobacteriota (average RA: 0.26%). Specifically, Firmicutes exhibited a significantly lower RA in Group FZ, whereas Proteobacteria showed a significantly higher RA in Group FZ compared to the other three groups (*p* < 0.05). Upon prolonged storage, Firmicutes RA (ranging from 91.86 to 99.39%) increased steadily, while Proteobacteria RA (ranging from 7.60 to 0.22%) decreased sharply. This inverse relationship—characterized by increasing Firmicutes dominance and declining Proteobacteria abundance—highlights a pronounced shift in the bacterial community structure during extended Yuba storage, indicating that storage duration exerted a significant influence on the phylum-level composition.

At the genus level (Figure 3C), the predominant genera (those with a relative abundance (RA) > 1%) included *Bacillus* spp., *Enterococcus* spp., *Lysinibacillus* spp., *Macrococcus* spp., *Leuconostoc* spp., *Lactococcus* spp., *Acinetobacter* spp., *Streptococcus* spp., and *Weissella* spp. In intergroup comparisons, Group FZ (7-day storage) exhibited significantly higher RAs of *Macrococcus* spp., *Leuconostoc* spp., *Lactococcus* spp., *Acinetobacter* spp., *Streptococcus* spp., and *Weissella* spp. (*p* < 0.01) compared to the other three groups (C, B, and A). Conversely, Group FZ had a significantly lower RA of *Bacillus* spp. (*p* < 0.01) relative to these groups. Notably, Group A (460-day storage) displayed a distinct bacterial profile, with a significantly higher RA of *Lysinibacillus* spp. (52.33%) than all other groups (*p* < 0.01). With prolonged storage duration, marked shifts in the RA of specific genera were observed. The RA of *Lysinibacillus* spp. increased significantly, rising from 0.27 to 52.33%. In contrast, the RA of *Macrococcus* spp. decreased significantly, dropping from 24.54 to 0.08%. These trends underscore the strong influence of storage time on the dynamics of the dominant bacterial genera in Yuba. Furthermore, short-term storage (7 days, Group FZ) is typified by higher abundances of several lactic acid bacteria (e.g., *Leuconostoc* spp., *Lactococcus* spp., *Weissella* spp.) and other genera (*Macrococcus* spp., *Acinetobacter* spp., *Streptococcus* spp.), whereas long-term storage (≥50 days) promotes the dominance of *Bacillus* spp., and *Lysinibacillus* spp. This shift likely represents adaptive changes in microbial communities in response to prolonged storage conditions.

### 3.4 Differential taxa analysis highlights storage-specific microbial enrichment

**Figure 4:**
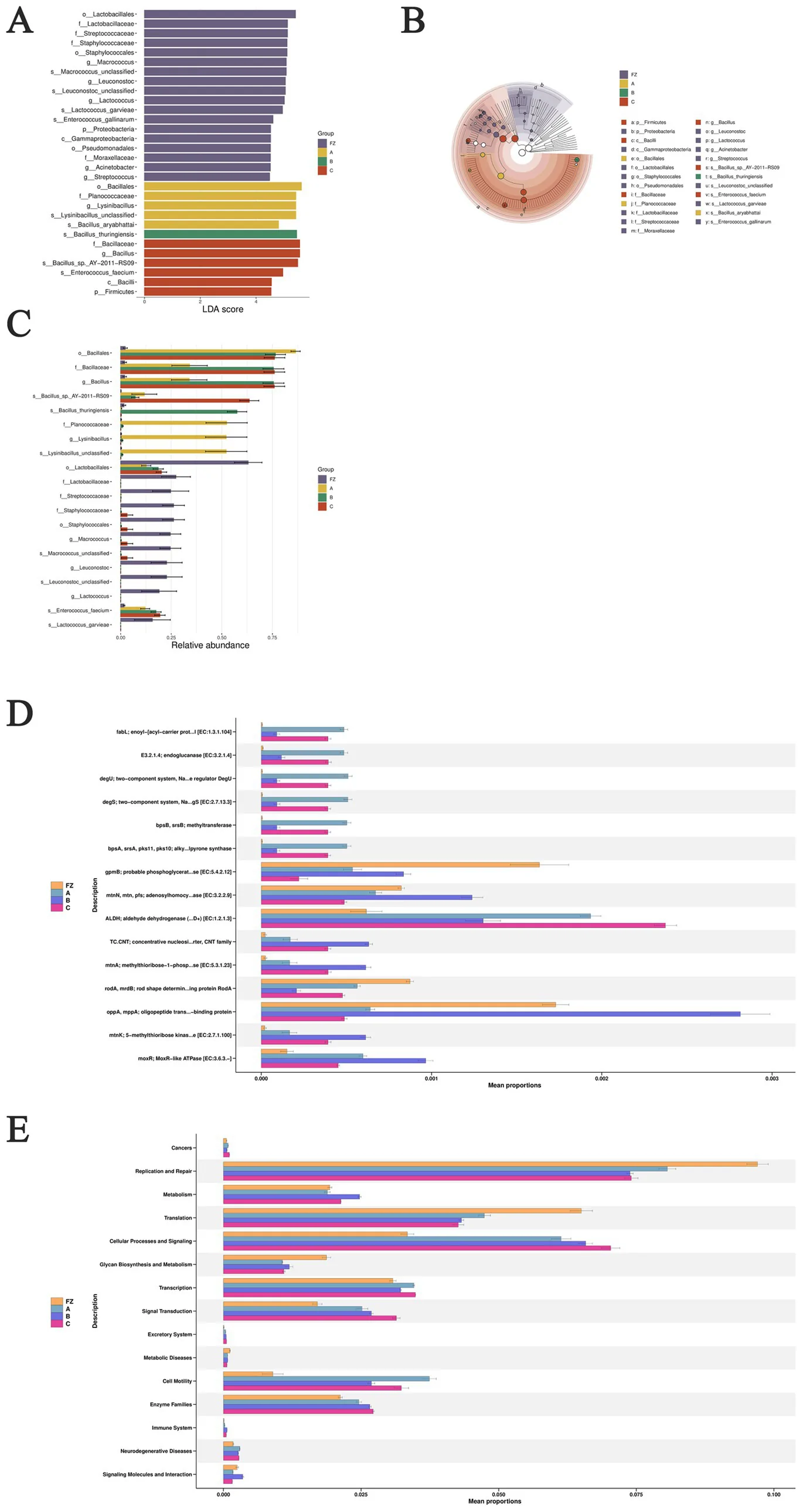
Differential Abundance of Microbial Taxa and Predicted Functional Potential During Yuba Storage. **(A)** Linear discriminant analysis (LDA) effect size (LEfSe) plot of significantly differentially abundant taxa (LDA score > 3.0, *p* < 0.05). Colors indicate the group with the most abundant taxon,and bar length represents the LDA score. **(B)** Taxonomic cladogram showing microbial community structure from kingdom to species. Node size corresponds to the relative abundance of the taxon. **(C)** Cladogram highlighting significantly differentially abundant taxa across the four storage durations. **(D, E)** Predicted functional profiles of the Yuba microbial community inferred using PICRUSt2. The *x*-axis represents the proportion of predicted functions. Functions are ordered by increasing *p*-values.

To gain a more detailed understanding of how storage duration influences the Yuba microbiota, LEfSe analysis was conducted to identify taxa with significant differences across groups, spanning phylum to genus levels. LDA results revealed that Group FZ (7 days storage) was significantly enriched in a diverse array of taxa, including Proteobacteria, Gammaproteobacteria, Lactobacillales, Staphylococcales, Pseudomonadales, Bacillales, Lactobacillaceae, Streptococcaceae, Staphylococcaceae, Moraxellaceae, *Macrococcus* spp., *Leuconostoc* spp., *Lactococcus* spp., *Acinetobacter* spp., and *Streptococcus* spp., reflecting a higher microbial diversity within this group (Figures 4A–C). In contrast, Group A was characterized by the enrichment of Bacillales, Planococcaceae, and *Lysinibacillus* spp. Group C, meanwhile, showed enrichment of Firmicutes, Bacilli, Bacillaceae, and *Bacillus* spp. Group B was unique in that it only had *Bacillus thuringiensis* enriched. These results reinforce the conclusion that storage duration significantly impacts the richness of the Yuba microbiota. Collectively, our findings demonstrate that storage duration distinctly affects both the richness and composition of the Yuba microbiota: shorter storage periods (e.g., Group FZ, 7 days) foster a more diverse microbial community, whereas longer or intermediate storage durations (Groups A, B, C) tend to select for specific, narrower taxonomic groups, with Group B being exclusively dominated by *B. thuringiensis*. This further highlights the pivotal role of storage duration in shaping the microbial community profile of Yuba.

Predicting microbiota function using PICRUSt2 based on 16S rRNA gene amplicon sequences reveals the functional potential of the microbial community. Figure 4D highlights that the most abundant predicted gene functions correspond to several key proteins with distinct biological roles: phosphoglycerate mutase, adenosylhomocysteine nucleosidase, NADP-dependent aldehyde dehydrogenase, rod shape-determining protein RodA, and oligopeptide transport system substrate-binding protein. Furthermore, Figure 4E identifies 39 differentially enriched pathways, underscoring major functional categories that together reflect the adaptive and metabolic prowess of the microbiota. These include replication and repair, metabolism, translation and transcription, cellular processes and signaling, signal transduction, cell motility, and enzyme families. These findings collectively indicate that the predicted microbiota functions are focused on core metabolic activities, structural maintenance, nutrient acquisition, and adaptive responses. Such functional orientation likely contributes to the microbial community’s ecological fitness and its functional interplay with the Yuba food matrix.

### 3.5 Correlation analysis of significant differential microbiota and metabolites in Yuba

Spearman correlation analysis was performed on the top five genera (ranked by relative abundance) to assess correlations between the significant differential microbiota and metabolites in Yuba during storage, and the resulting heatmap illustrated significant correlations.

**Figure 5:**
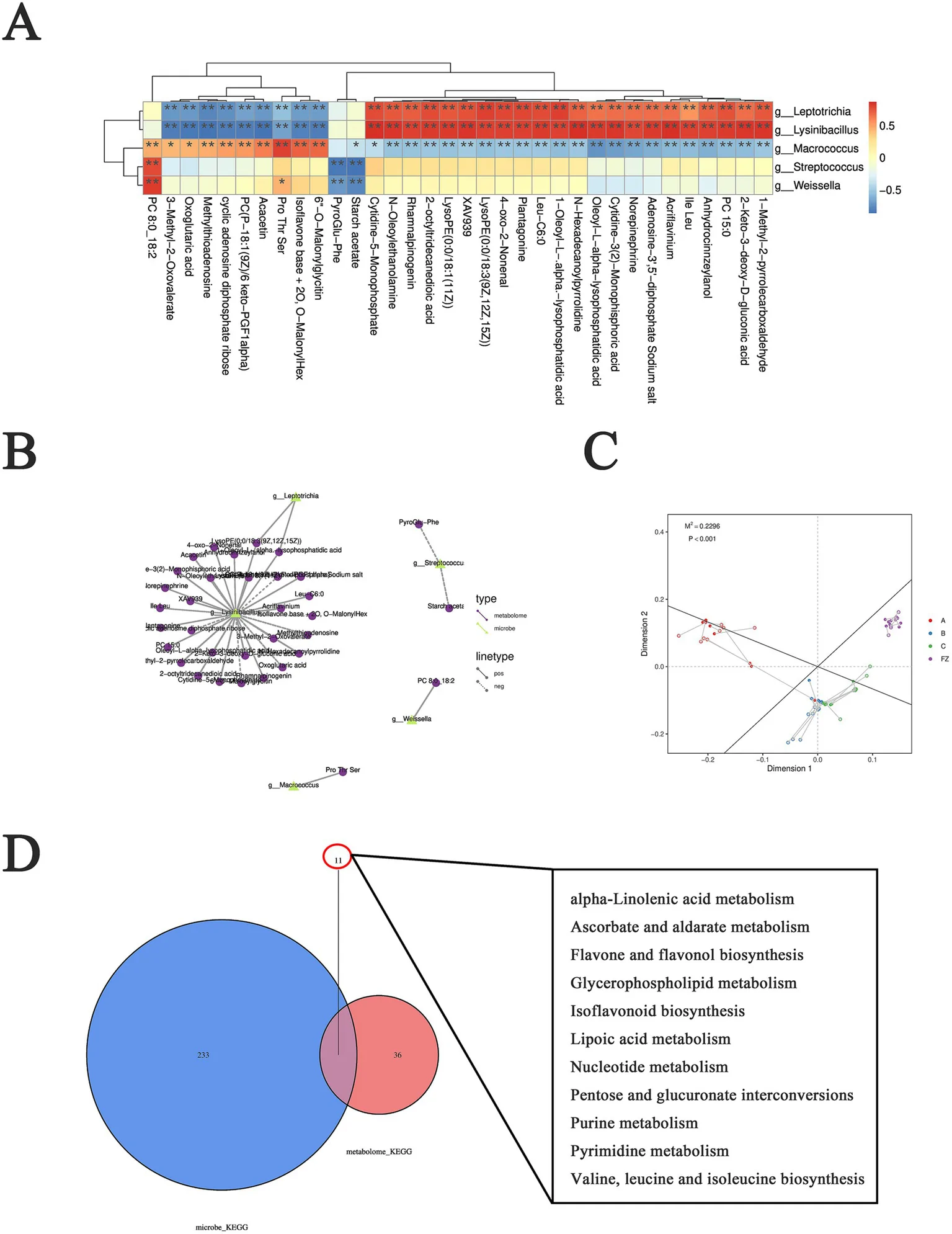
Correlation analysis reveals relationships between microbial communities and metabolite profiles in Yuba. **(A)** Correlation heatmap displaying Spearman correlations between significantly altered microbial genera and metabolites. Red indicates positive correlation, and blue indicates negative correlation. Asterisks denote significance levels (* *p* < 0.05; ** *p* < 0.01). **(B)** Network diagram illustrating correlations between altered metabolites (purple circles) and bacterial genera (green triangles). Solid lines indicate positive correlations, and dashed lines indicate negative correlations. **(C)** Procrustes analysis comparing microbial community and metabolomic data. Colors represent different storage durations. Each line connects a sample’s microbial community data point (open dot) to its corresponding metabolomic data point (solid dot). *M*2 represents the statistic of the *M*2 statistic. The *p*-value was determined by Monte Carlo simulation. **(D)** Venn diagram showing the overlap of significantly enriched KEGG pathways (*p* < 0.05) identified in single-omics analysis.

The abundances of *Lysinibacillus* spp. were significantly positively correlated (*p* < 0.01) with the levels of 1-methyl-2-pyrrolecarboxaldehyde, 2-keto-3-deoxy-D-gluconic acid, PC 15:0, anhydrocinnzeylanol, Ile-Leu, acriflavinium, adenosine-3′,5′-diphosphate sodium salt, norepinephrine, cytidine-3(2)-monophosphoric acid, 1-oleoyl-L-*α*-lysophosphatidic acid, Leu-C6:0, plantagonine, 4-oxo-2-nonenal, LysoPE(0:0/18:3(9Z, 12Z, 15Z)), XAV939, LysoPE(0:0/18:1(11Z)), 2-octyltridecanedioic acid, rhamnalpinogenin, N-oleoylethanolamine, and cytidine-5-monophosphate (Figures 5A,B). Conversely, their abundances were significantly negatively correlated (*p* < 0.01) with levels of 6”-O-malonylglycitin, isoflavone base + 2O, O-malonylHex, Pro-Thr-Ser, acacetin, PC(P-18:1(9Z)/6keto-PGF1*α*), cyclic adenosine diphosphate ribose, methylthioadenosine, oxoglutaric acid, and 3-methyl-2-oxovalerate. This correlation pattern was reversed in *Macrococcus* spp. These findings underscore divergent metabolic associations between *Lysinibacillus* spp. and *Macrococcus* spp., indicating potential functional differences in their roles in shaping the metabolite profile of Yuba.

Additionally, the relative abundances of *Streptococcus* spp. and *Weissella* spp. showed a significant negative correlation (*p* < 0.01) with starch acetate and pyroGlu-Phe levels, and a significant positive correlation (*p* < 0.01) with PC 8:0_18:2 levels. These results highlight the complexity of microbial-metabolite interactions, which may be critical for understanding microbiome functionality in Yuba-associated contexts. Procrustes analysis revealed that the association between bacterial communities and metabolites in Group FZ was significantly closer than in the other three groups (*p*-value = 0.001, M^2^ = 0.2296; Figure 5C). The closer association in Group FZ relative to the other three groups carries important implications for understanding the interplay between microbiota and metabolism in this context. Future investigations should focus on identifying specific bacterial taxa or metabolic pathways driving this association, as well as exploring environmental or experimental factors unique to Group FZ that foster such tight coupling.

Next, Venn diagram analysis revealed 11 pathways common to significantly altered microbes and metabolites in Yuba, including α-linolenic acid metabolism, ascorbate and aldarate metabolism, flavone and flavonol biosynthesis, glycerophospholipid metabolism, isoflavonoid biosynthesis, lipoic acid metabolism, nucleotide metabolism, pentose and glucuronate interconversions, purine metabolism, pyrimidine metabolism, and valine, leucine, and isoleucine biosynthesis (Figure 5D). The significant pathways overlap between differential microbes and metabolites provide compelling support for a model wherein dynamic shifts in the microbial community during storage enable it to actively regulate Yuba’s metabolic landscape. By acting through these pathways, microbes likely drive changes in lipids, amino acids, nucleotides, polyphenols, and carbohydrates, ultimately shaping Yuba’s nutritional properties, flavor, and shelf-life stability. This insight underscores the importance of microbial-metabolite interactions as a critical determinant of Yuba quality during storage.

## 4 Discussion

Our study, integrating 16S rDNA sequencing and LC–MS metabolomics, provides a systems-level understanding of the complex interplay between microbial community shifts and metabolite alterations driving Yuba spoilage during ambient storage. These findings offer a robust foundation for assessing Yuba quality over time and pave the way for targeted preservation strategies.

Our results highlight dynamic remodeling of the Yuba metabolome with prolonged storage, notably through elevations in lipids, fatty acyls, and amino acid-related metabolites, coupled with selective depletions in certain compounds. The dominance of lipids (16/30 of the most abundant metabolites), particularly the increased levels of glycerophospholipids (LysoPEs, LysoPA, PCs) and fatty acyls (Methyl-9-hydroperoxy-delta10E,12Z-octadecadienoate, 2-octyltridecanedioic acid), is a key finding. These increases are consistent with enzymatic degradation of membrane phospholipids, likely by bacterial phospholipases, or oxidative damage, as LysoPE and LysoPA accumulation impairs membrane stability, potentially reducing Yuba’s water-holding capacity and contributing to textural degradation (11, 30). While Bi et al. (11) demonstrated similar phospholipid degradation using GC-IMS, our UPLC–MS/MS approach enabled a more comprehensive identification of specific lysophospholipid species and their temporal dynamics over extended ambient storage, not previously reported. Furthermore, the rise of Methyl-9-hydroperoxy-delta10E,12Z-octadecadienoate, an oxidation product of linoleic acid, confirms ongoing oxidative rancidity, diminishing flavor quality and nutritional value (31).

Concurrently, we observed an increasing trend in amino acid (AA)-related metabolites (L-tyrosine, Pro-Ile-Ile, Methionine sulfoxide, etc.) during storage. Given Yuba’s high protein content, this accumulation suggests proteolytic activity, possibly by endogenous proteases or more likely by Bacillus and *Lysinibacillus* spp. microbial proteases under ambient conditions. While initial accumulation of these nitrogenous compounds could contribute to umami taste, excessive hydrolysis can lead to texture softening and bitter off-flavors, particularly through hydrophobic peptides such as Prolylhydroxyproline (32). The presence of Methionine sulfoxide, indicating amino acid oxidation, and *γ*-Glutamylleucine, suggests amino acids undergo oxidative or metabolic modifications during storage depleting essential amino acids and diminishing Yuba’s nutritional value (33–35). While Qiu et al. (36) investigated frozen storage conditions, our study identifies key metabolites associated with ambient room temperature storage, broadening the known influence of storage temperature on metabolites.

Microbial community analysis revealed that Firmicutes dominated across all storage groups, with a shift toward increasing abundance of Firmicutes and decreasing Proteobacteria as storage duration increased. This aligns with findings in other anaerobically packaged foods (37, 38). Li et al. (10) previously established the dominance of *Bacillus* spp. in Yuba due to the heat resistance of their spores, our data shows the emergence of *Lysinibacillus* spp. after prolonged storage.

Notably, the most striking change in microbial composition was the significant increase in *Lysinibacillus* spp. relative abundance (RA) from 0.27 to 52.33% with extended storage (460 days). Although *Lysinibacillus* spp. are environmentally widespread (39) and some species can be used in biocontrol against plant pathogens (40), some poses risk to food storage due to heat-resistant spores (41), its presence in Yuba and its relationship to spoilage have not been previously described. The strong positive correlation we observed between *Lysinibacillus* abundance and lipid degradation metabolites (e.g., glycerophosphocholine) suggests a role as a key driver of spoilage, possibly through the action of extracellular lipases, though this proposed mechanism requires confirmation through pure culture studies. Specifically, since our study determined a statistical correlation, future research should attempt to create a pure culture of *Lysinibacillus* to confirm the findings and analyze what enzymes the bacteria produce.

Conversely, the RA of *Macrococcus* spp. was strongly negatively correlated with glycerophospholipid levels and strongly positively correlated with 6”-O-malonylglycitin levels. This inverse relationship suggests that *Macrococcus* may be associated with a slower accumulation of lipid degradation products. This correlation between *Macrococcus* spp. and 6”-O-malonylglycitin, coupled with its increased prevalence in freshly prepared Yuba (Group FZ), indicates its potential contribution to isoflavone malonation in Yuba and its positive effect on bioavailability (42). The effects of *Macrococcus* on isoflavone malonation could be used in pure-culture studies.

Among lactic acid bacteria (LAB), *Weissella*, *Leuconostoc*, and *Lactococcus* spp. exhibited decreased RA with extended storage, indicating that the conditions in sealed Yuba packages are not favorable for their growth. These observations support the dual roles of these bacteria in food storage; some species have potentially beneficial effects, and other contribute to spoilage (43, 44).

The study identified 11 common pathways between significantly differential microbes and metabolites, further suggesting that microbial activity actively regulates Yuba’s metabolic landscape during storage. These encompass lipid metabolism, phytochemical composition, nucleotide turnover, and carbohydrate metabolism in Yuba. By targeting these pathways, microbes likely drive changes in lipids, amino acids, nucleotides, polyphenols, and carbohydrates, shaping Yuba’s nutritional properties, flavor, and shelf-life stability.

In conclusion, this research provides valuable insights into the interplay of metabolites and bacterial communities during long-term Yuba storage. Our findings demonstrate the importance of lipid degradation, the potential of *Lysinibacillus* spp. as a key spoilage agent, and the correlation between microbial activity and Yuba’s metabolomic profile. Future research, combining *in vitro* co-cultivation experiments, and metatranscriptomic analyses, should further investigate regulatable microbial enzymes (e.g., lipases, proteases) and regulatory mechanisms. Such knowledge will contribute to developing targeted strategies for quality control, preservation, and enhancement of the overall value and consumer acceptance of Yuba.

While this study provides a detailed characterization of spoilage in Yuba from a single production source, several limitations should be acknowledged.

Generalizability of Biomarkers: Although our findings offer valuable insights into Yuba spoilage within a specific production context, the identified microbial and metabolic signatures may not be universally applicable to all Yuba products. Variations in production processes and raw material sourcing across manufacturers and regions can lead to distinct microbial communities and metabolic profiles. Future studies should aim to validate these findings using Yuba samples from multiple producers and geographic locations to identify core spoilage indicators.

Causative Role of *Lysinibacillus*: Our study established a strong statistical correlation between the abundance of *Lysinibacillus* spp. and specific spoilage-associated metabolites. However, correlation does not imply causation. To definitively establish *Lysinibacillus* as a primary spoilage agent, further research involving controlled inoculation studies with isolated *Lysinibacillus* strains is necessary. Such studies should include analyses of the specific enzymes produced by these strains and their impact on Yuba quality under defined conditions.

Scope of Metabolomic Analysis: Although UPLC-HRMS enabled comprehensive profiling of non-volatile metabolites, it did not capture volatile compounds, which are key contributors to Yuba’s aroma and spoilage perception. Previous studies have identified volatile aldehydes and alcohols as important aroma markers (11). Future research would benefit from incorporating volatile analysis (e.g., using GC–MS) to provide a more holistic understanding of Yuba spoilage.

Predictive Microbial Functionality: The PICRUSt2 analysis provided predictions of the functional potential of the microbial community. While informative, these predictions are based on genomic data and do not reflect actual gene expression or metabolic activity under the specific storage conditions. Metatranscriptomic or metaproteomic approaches would offer a more direct assessment of microbial functions *in situ*.

Single Production Batch: All Yuba samples originated from a single production batch. While this approach ensured consistency and minimized batch-to-batch variability within the study, it limited the exploration of potential variations arising from different production conditions. Additionally, we acknowledged Yuba’s growing market presence and the lack of readily available detailed statistics, while emphasizing its importance as both a traditional and emerging plant-based protein source. Therefore, future research could explore these market dynamics to further highlight the practical implications of effective spoilage management. This systematic approach will help distinguish between universal spoilage indicators and those specific to particular production environments. The proposed mechanisms are supported by strong correlational evidence and warrant further experimental validation.

## 5 Conclusion

This study, employing comprehensive metabolomic and microbial analyses of Yuba samples stored at room temperature for varying durations (7, 50, 105, and 460 days), reveals a dynamic and interconnected relationship between the Yuba metabolome and microbiota that evolves significantly during storage. Our findings demonstrate that storage duration is a critical factor influencing both the microbial community composition and the metabolomic profile, with lipid and amino acid metabolism being particularly susceptible to alteration. Moreover, the dominance of *Lysinibacillus* and its significant correlations with spoilage-associated metabolites highlight it as a key candidate contributing to quality deterioration. Controlling the growth of this genus, along with other dominant Firmicutes such as *Bacillus*, is likely important for extending shelf-life. Future work using sterile Yuba models inoculated with individual strains will be necessary to definitively establish their specific roles in spoilage.

## Data Availability

The original sequence data for 32 Yuba samples are available at the NCBI SRA under the accession number PRJNA1345171 (https://www.ncbi.nlm.nih.gov/sra/PRJNA1345171).
